# The Long Road to Recovery: Environmental Health Impacts of Hurricane Sandy

**DOI:** 10.1289/ehp.121-a152

**Published:** 2013-05-01

**Authors:** John Manuel

**Affiliations:** John Manuel of Durham, NC, is a regular contributor to *EHP* and the author of *The Natural Traveler along North Carolina’s Coast* and *The Canoeist*.

Building contractor John Pierciey stands in the gutted interior of a 1950s-era home in Manasquan, New Jersey. Wallboard, two layers of wood flooring, a layer of felt—all of it had to be ripped out to rid the house of mold caused by Hurricane Sandy’s storm surge. “This is the sixth house I’ve gutted in a week,” Pierciey says. “Every one is different. You don’t know what you’re going to find until you take them apart.”

**Figure:**
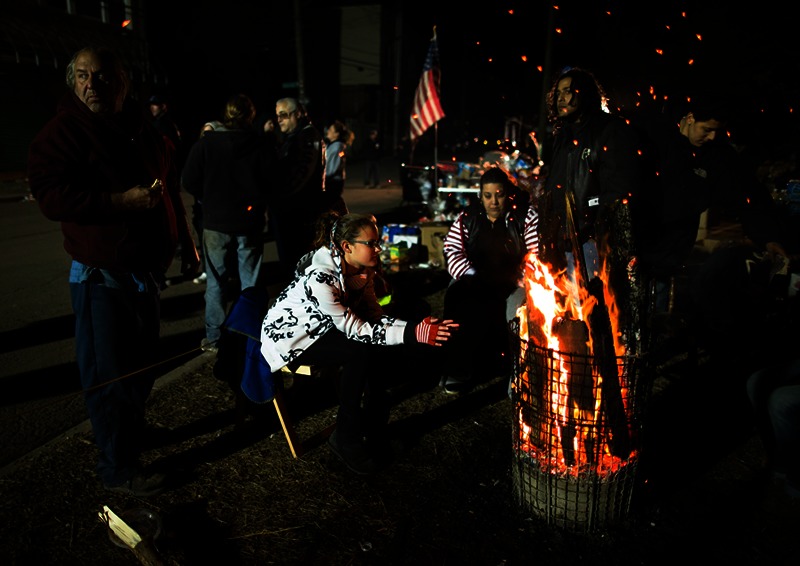
Residents and volunteers keep warm by a fire barrel in the New Dorp Beach neighborhood of Staten Island, 5 November 2012. Power outages persisted through a cold snap that put temperatures in the single digits. © AP Photo/John Minchillo

So it is with the environmental health impacts of Hurricane Sandy. Every layer of society, every type of building, has felt the impact of the storm, which struck the U.S. East Coast on 29 October 2012. Incidences of death and illness, though small in number compared with some storms, have come in many forms, the effects still unfolding as time goes by.

## Immediate Impacts

Hurricane Sandy was the largest storm ever recorded in the Atlantic Ocean. It reached more than 1,000 miles in diameter and affected states from Florida to Maine.^1^ Sandy was responsible for an estimated 234 deaths in 8 countries and caused potentially $50 billion in property damage in the United States alone.^1^ In New York and New Jersey alone, the storm damaged or destroyed more than 375,000 housing units.^2^ Months after the storm, power had still not been restored to all areas, and access to towns on the New Jersey barrier islands was limited to contractors and homeowners, and then only during daylight hours. (As of this writing, power has been restored to all customers.)

In terms of immediate impact, the greatest health threat came from the storm surge that swept into densely populated communities along the New Jersey shore, Long Island, and Lower Manhattan. The storm’s arrival coincided with a high tide to push onshore a destructive surge of water 12.5 feet high at its peak.^1^ Of the 97 deaths recorded in the New York metropolitan area—which includes northern New Jersey and parts of Connecticut—most were from drowning.^3^

**Figure:**
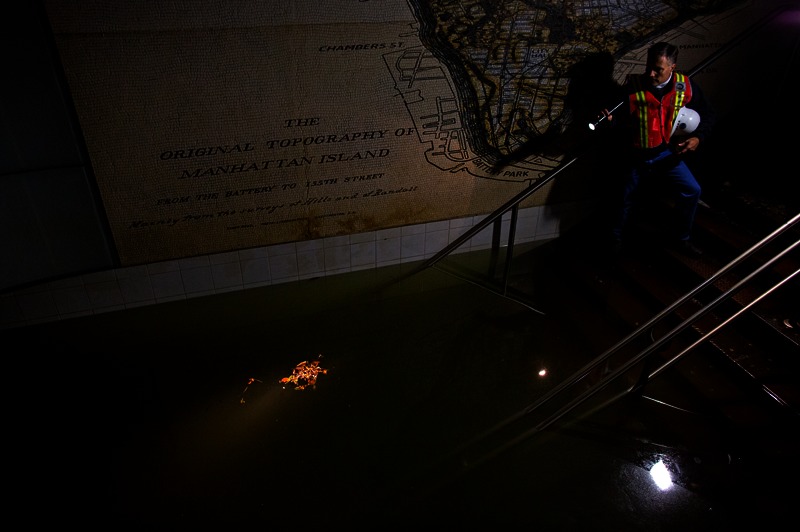
Joseph Leader, vice president and chief maintenance officer of the Metropolitan Transportation Authority, shines a flashlight on standing water inside the South Ferry 1 train station in New York, 31 October 2012. Subway stations and tunnels throughout Lower Manhattan were flooded by Sandy’s storm surge. A portion of the Community Development Block Grant Disaster Relief funds authorized in January 2013 will go toward investments in infrastructural resilience against future disasters. © AP Photo/Craig Ruttle

Fire posed another hazard. After seawater short-circuited the electrical system in a house in New York’s Breezy Point, wind-swept flames spread to 126 homes. Dozens of fires broke out in other areas as a result of the storm. Amazingly, there were no deaths from any of these.^4^

Astute preparations in advance of the storm saved countless lives. Unlike with Hurricane Katrina, which seemed to capture leaders at all levels unprepared, a wide net of government agencies was on hand to deal with the health and security threats posed by Hurricane Sandy. The National Guard deployed 200 troops to keep order in New York City. The Federal Emergency Management Agency (FEMA) sent Incident Management Assistance Teams to coordinate federal resources to support the states. The U.S. Coast Guard positioned teams along the coast for search and rescue. The Nuclear Regulatory Commission maintained watch over nuclear plants, three of which were shut down during the storm. New York City mayor Michael Bloomberg and New Jersey governor Chris Christie ordered the evacuation of some coastal areas as well as the closure of bridges and tunnels throughout the metropolitan area, along with subway lines, commuter trains, bus lines, and the three major airports. These closures proved prescient as all road tunnels into Manhattan, except the Lincoln Tunnel, were subsequently flooded, as were subway stations and tunnels in Lower Manhattan.

Immediately following the storm, another wave of agencies sprang into action. The U.S. Department of Health and Human Services deployed more than 500 personnel, including 9 Disaster Medical Assistance Teams from 8 states, to provide care at medical shelters across the area. The American Red Cross opened 171 shelters across 13 states, with thousands of volunteers working alongside paid personnel. FEMA set up 68 Disaster Recovery Centers in Connecticut, New York, and New Jersey where people could apply for assistance and seek information on alternative housing. Within 24 hours, FEMA supplied more than a million liters of water and more than a million shelf-ready meals to the New York National Guard and to volunteers to distribute to those in need.^5^

## Loss of Power

In the days and weeks after the storm, the greatest public health threat was from the loss of power. Sandy knocked out electricity for more than 8.5 million people in 21 states.^6^ This loss of power, coupled with the absence or flooding-related failure of backup generators, translated to the shutdown of heating systems, life support, and other technologies that were vital to people’s survival. More than 1,000 patients had to be evacuated from New York metro area hospitals, including New York University’s Langone Medical Center, Bellevue Hospital, Coney Island Hospital, and Palisades Medical Center. This was accomplished without any reported losses of a human life.

Research animals were not so lucky. At New York University’s Smilow Research Center, 10,000 lab rats being used for long-term research drowned when floodwaters inundated the basement.^7^ These animals had been specifically bred over a period of years to model human diseases and disorders including cancer, autism, epilepsy, and heart disease. “It’s so horrible, you don’t even want to think about it,” center cancer biologist Michelle Krogsgaard told *ABC News* of the loss of the animals. “All the work we did, all the time and money, we’re going to have to start over.”^8^

Loss of power presented a distinct threat to people living in the region’s many high-rise apartments. In normal times, those living on upper floors consider themselves lucky to enjoy the views. But when the electricity went out in these buildings, the elevators stopped working, and many of those same people—physically unable to descend the stairways—were trapped for days and even weeks on end.

Nastaran Mohit is a volunteer for Occupy Sandy, a nonprofit group coordinating relief efforts to victims of the hurricane. In the days following the storm, she established a “pop-up” medical clinic in the Rockaways that recruited dozens of volunteer doctors, nurses, and mental health professionals to aid the storm victims. The Rockaways is a densely populated peninsula fronting the Atlantic Ocean that was completely inundated with floodwater. It is home to four major public housing projects as well as a number of nursing homes and halfway houses. In the week after the storm, Mohit began sending teams to search out and help residents of these high-rises.

“What they found was frightening,” Mohit says. “There were literally thousands of elderly people trapped in the upper floors of these buildings. The hallways were pitch black. Many apartments were without functioning plumbing. People were living in their own feces.”

**Figure:**
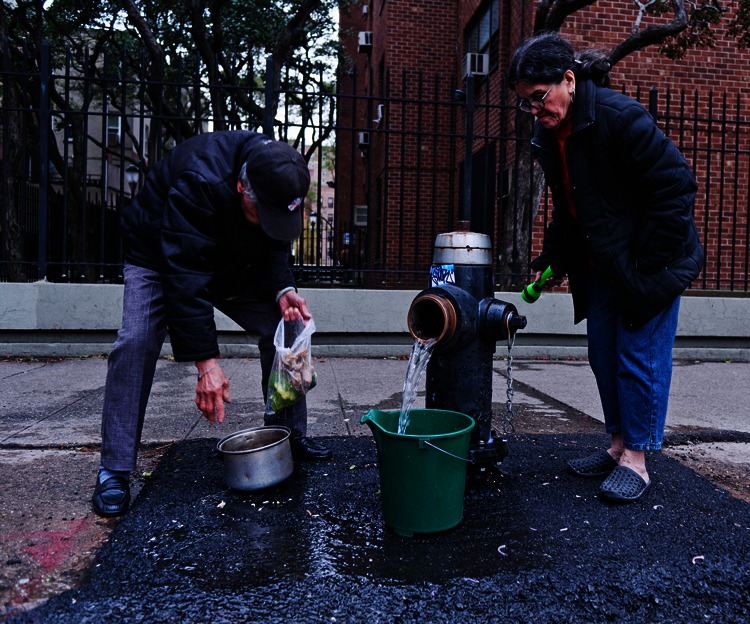
A couple collects water from a fire hydrant on Manhattan’s Lower East Side, 2 November 2012. © Angelo Merendino/Corbis

**Figure:**
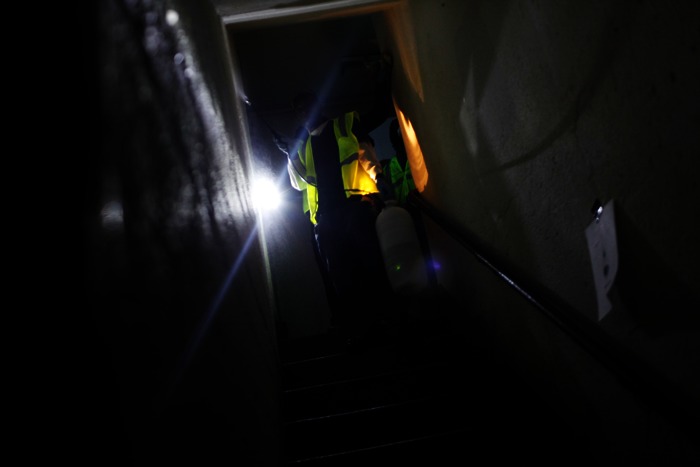
Despite the ongoing lack of power, cleanup must proceed; here, a New York City housing worker cleans and disinfects a Red Hook home, 13 November 2012. The storm knocked out power to more than 8 million people, and prolonged loss of electricity and running water posed a significant problem for many people. © Andrew Lichtenstein/Corbis

Mohit says many elderly residents suffered from chronic medical conditions—arthritis, high blood pressure, and diabetes. As time stretched on, she says, “We found residents who had gone for weeks without pain or cancer medication. They had to ride it out until we could send runners out to get the proper meds. Even then, a lot of pharmacies wouldn’t honor the prescriptions because we didn’t have exactly the right information for them to get insurance coverage.”

Two months after the storm, thousands of residents in the Rockaways high-rises were still without functioning elevators. Governmental and private agencies are now providing aid, but volunteers like Mohit continue to fill what they see as a gaping void in the disaster response system. “There is a misconception that if a major storm hits, someone is going to take care of you,” Mohit says. “It’s just not true.”

Every storm in which power is lost for an extended period of time seems to result in tragedies related to carbon monoxide (CO) poisoning. People resort to using gas stoves or ranges to heat their dwellings and portable gas generators to provide homes with electricity. The situation was exaggerated after Sandy, given the sheer number of gas generators purchased and the threat to safety that presented.^9^

“Before the hurricane none of my neighbors had generators, but after Sandy they all did,” says Brian Buckley, a resident of Sea Girt, New Jersey, and executive director of laboratories at the Environmental and Occupational Health Sciences Institute (EOHSI) in Piscataway. Buckley knew his neighbors were running their generators properly because he could hear them running as he walked down the street—“You would only hear them running if they were outside,” he explains.

But not everyone in the affected area knew how to properly use a generator, Buckley says; some ran them in their garages and inside their homes, creating hazardous CO levels. And Paul Lioy, deputy director of EOHSI, points out, “The proliferation of devices in neighborhoods will change the magnitude of the local CO exposure issue during the next blackout.”

**Figure:**
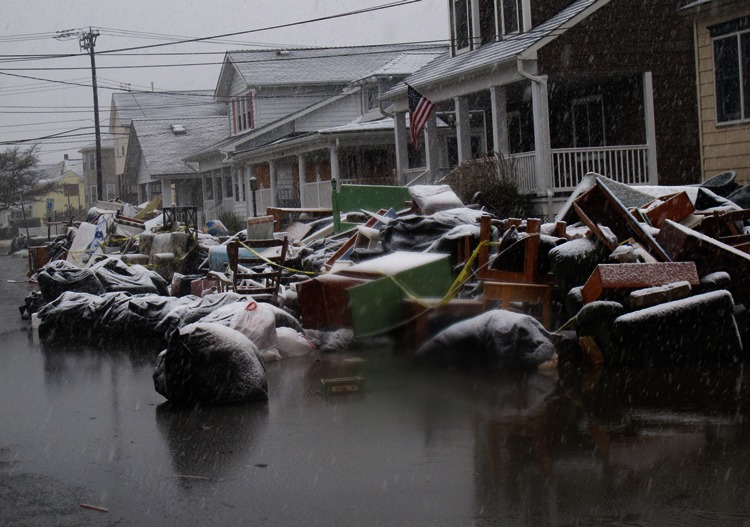
Point Pleasant Beach, New Jersey, 7 November 2012. AP Photo/Wayne Parry

**Figure:**
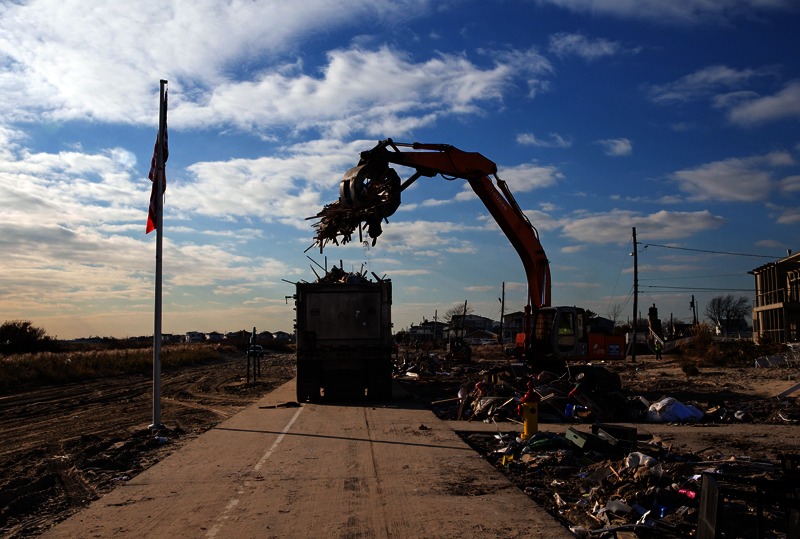
The Breezy Point neighborhood of Queens, New York, 14 November 2012. Hurricane Sandy was the second costliest storm to hit the United States, with damages potentially reaching as high as $50 billion.1 According to FEMA, 95% of the 5.25 million cubic yards of debris generated by the storm had been removed by February 1, but demolition of destroyed structures was only 20% complete.^27^ © Scott Houston/Corbis

The Centers for Disease Control and Prevention (CDC) had, as of November 6, collected 263 reports of CO exposure from poison control centers—a figure that included 4 deaths—and suspected there might be more.^10^ By November 17 local authorities were reporting 8 deaths as known or suspected to be caused by CO poisoning.^11^

## Outdoor Air and Water

Outdoor air quality becomes a concern after flooding events when sediment deposited by floodwaters on city streets and sidewalks dries and is kicked up by vehicles and foot traffic. Damaged buildings are demolished, and debris is stacked on sidewalks, trucked away, and sometimes burned. New Yorkers are particularly sensitive to the issue of air quality after initial monitoring by the EPA in the aftermath of 9/11 failed to detect hazardous particles from the collapse of the World Trade Center buildings.^12^ These particles included asbestos, lead, mercury, and crystalline silica, all of which are known to contribute to cancer or respiratory ailments.^13^

The New York Department of Environmental Conservation (DEC) routinely monitors air quality around the state to forecast Air Quality Indices. These monitors showed no overall increase in ambient air pollution after Sandy, but they were not located near areas where debris was being gathered for removal to landfills.^14^ So in December, DEC and the EPA set up three additional monitoring stations in areas hardest hit by Sandy. Those monitors showed measurements of fine particulate matter exceeding the EPA’s recommended 24-hour standard in several locations, including Lower Manhattan, but only on a few days.^15^ There were no exceedances of air quality in New Jersey, according to Jane Kozinski, assistant commissioner of environmental management with that state’s Department of Environmental Protection (NJDEP).

**Figure:**
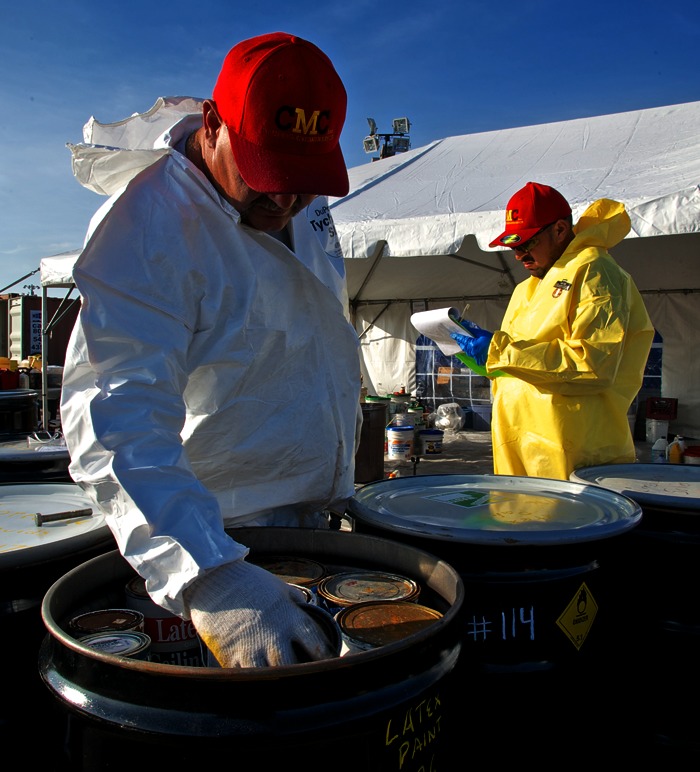
Hurricane Sandy swept hazardous chemical containers from homes and businesses and deposited them into nearby marshland. In this undated photo, a worker with the EPA prepares some of these orphaned household chemicals for disposal. Eliud Echevarria/FEMA

**Figure:**
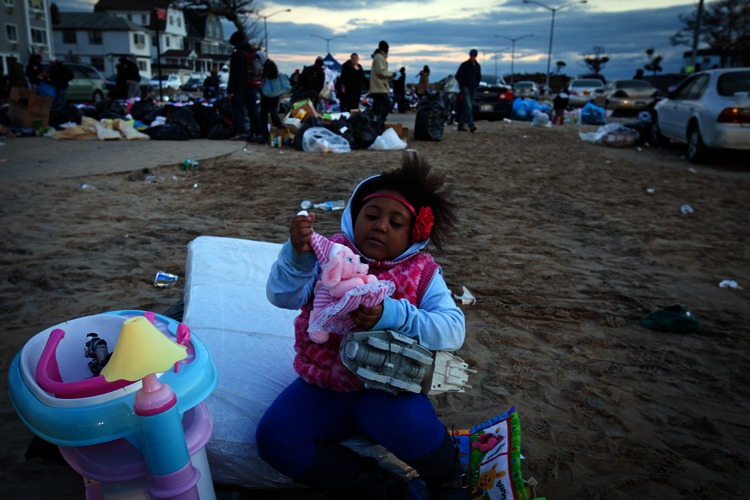
A child plays with donated toys while her family looks through other donated goods in Rockaway Beach, Queens, New York, 4 November 2012. Natural disasters bring out many people’s desire to help. However, poorly organized or inappropriate donations can cause a “second-tier disaster” as relief workers struggle to process a deluge of well-meant but unusable items.^28^ © Amy Sussman/Corbis

Water pollution was a major health concern after Hurricane Sandy. Raw sewage spilled into homes in Baldwin and East Rockaway, New York, when a sewage plant flooded and could not handle the volume.^16^ Jill Lipoti, director of the Division of Water Monitoring and Standards with the NJDEP, says the storm knocked out power or damaged some 80 sewage treatment systems in that state, including the Passaic Valley Sewerage Commission, one of the largest sewage treatment plants in the country. During wet weather, the plant treats up to 550 million gallons of sewage per day. Based on that figure, Lipoti estimates that as much as 2.75 billion gallons of untreated waste flowed from the plant into the nearby bay during the five days the plant was out of commission.

In the days after the storm, the state of New Jersey issued advisories for public recreational waters impacted by sewage. Shellfish waters were closed statewide. Boil-water advisories were issued for affected water supply systems. “If you lose power in your water supply system, you have to issue an advisory to the public asking them to boil their water before drinking because you don’t know if the water has been contaminated,” Lipoti says. All recreational and boil-water advisories have since been rescinded, and most shellfish waters have reopened.

In Lower Manhattan, stormwater flooded five subway tubes, two Amtrak tunnels, and three of the city’s primary roadways. FEMA assigned the pumping out of these structures to the U.S. Army Corps of Engineers (USACE) Unwatering Team, dubbed by the press as the “Unwatering SWAT Team.” In less than two weeks, this team pumped an estimated 275 million gallons of seawater from the major tunnels under New York City.^17^ Roger Less, chief of the Design Branch of the USACE Rock Island District, says New York City officials did an excellent job of vacating the tunnels prior to Sandy’s landfall, thereby addressing potential public safety issues while also minimizing water pollution that might have occurred from hydrocarbons escaping submerged vehicles.

## A Window of Opportunity

**Improving Energy Efficiency in Sandy’s Wake**

Hurricane Sandy is estimated to have damaged more than 375,000 homes, many of which have been gutted to remove water-damaged building components. This situation, anguishing as it is to homeowners, presents a unique opportunity to upgrade the energy efficiency of these buildings. Improving energy efficiency will not only reduce energy consumption but also reduce greenhouse gas emissions involved in producing that energy.^29^

Because of uncertainties regarding new FEMA regulations for flood insurance, many homeowners have yet to begin reconstruction of damaged or destroyed houses. However building and insulation contractors are reporting that of those who have begun work, many are choosing to add energy-efficient components.

**Figure:**
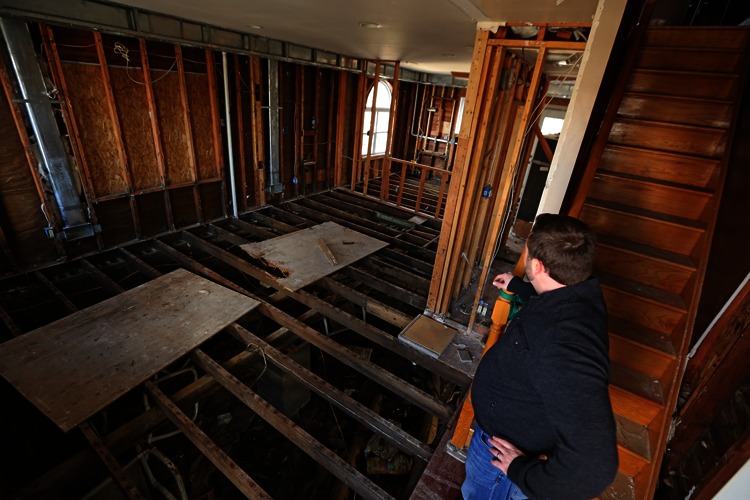
A home in Old Bridge Township, New Jersey, is stripped to the framing for cleanup and repairs. © Frank Conlon/Star-Ledger/Corbis

“Of my clients who are rebuilding, most seem to be upgrading energy efficiency,” says John Pierciey, a custom home builder in New Jersey. “As long as you’re going to [rebuild], you might as well do it right.”

Pierciey says the most popular improvements are closed-cell foam insulation sprayed into wall cavities and under floors, and high-efficiency gas furnaces to replace older furnaces destroyed by floodwaters. According to the U.S. Department of Energy, closed cell foam has a higher insulating value (R-value) per inch than fiberglass,30 and contractors claim that, properly installed, it is more effective at blocking air flow. Furthermore, it has a perm rating of 0.89, which effectively makes it a vapor barrier.31 Installed in the floor, it can seal off the living area from moisture in the crawlspace.

## Long-Term Considerations

Of the long-term health threats posed by Sandy, the most significant is mold growth in homes that were not properly remediated after flooding. Indoor exposure to mold has been linked to upper respiratory tract symptoms, cough, and wheeze in otherwise healthy people, and with exacerbation of symptoms in people with asthma.^19^ To rid a flooded house of mold, all wet furnishings and building materials composed in whole or in part of cellulose fiber—including wood flooring and wallboard—must be demolished and removed from the house. Wood framing must then be scrubbed free of mold with a detergent solution and dried using dehumidifiers and blowers before reconstruction begins. Nonporous surfaces affected by floodwater containing sewage can be cleaned with a dilute bleach solution, but Bill Sothern, a certified industrial hygienist with the firm Microecologies, says bleach should not be used on wood.

Following Sandy, an army of contractors and volunteers descended on the flood zones to offer their services in demolition and mold remediation. Interviews with volunteer groups and contractors for this article suggest that most workers were instructed to use respirators when working in these spaces. However, Sothern observed that many workers chose not to follow this recommendation. More significantly, he says, not all mold was removed in these remediation efforts, which could present problems down the road.

Sothern says virtually every one of the 200 flood-damaged homes his company examined prior to remediation had substantial levels of visible mold growth on the underlying structural wood components (studs/sills), which was revealed when the wet wallboard was removed. Similarly, mold growth on the top and bottom sides of subflooring and on the underlying structural floor joists is a ubiquitous problem. Removing structural components is very expensive, not to mention impractical to perform in the cold of a Northeast winter, and thus has not been common practice for homes flooded by Sandy, according to Sothern. Even if the mold is cleaned from the accessible surfaces, that means some moldy material is bound to remain.

“Under the best of circumstances, if the moldy wood is cleaned and dried out before rebuilding, there will still be mold left in the inaccessible wood-to-wood interfaces,” Sothern says. “And in many cases we know that the homes are being rebuilt without either properly cleaning or drying out the affected wood.”

The impact of these scenarios on airborne mold levels is not well understood, so Sothern—who is also a doctoral candidate at the City University of New York School of Public Health—and a team at the university have designed a study to examine these associations. The Respiratory Health Effects and Exposure to Mold study would inspect 300 homes in the Rockaways and collect data on visible mold growth conditions, moisture conditions, remediation/rebuild status, and airborne mold levels, and interface these data with the responses to a respiratory health questionnaire. As of this writing, the study has yet to be funded.

“The associations that are found to exist between the environmental conditions and the respiratory health conditions will answer many questions that can help to inform best practices for effectively remediating mold growth on structural wood that frequently occurs as a result of flooding,” Sothern says.

Ongoing measurements of mold in New Jersey homes are being conducted by a team of environmental health experts from Rutgers University, including Lioy and Buckley of EOHSI, Joan Bennett of the Office for the Promotion of Women in Science, Engineering and Mathematics, and Gediminas Mainelis of the School of Environmental and Biological Sciences. They have been sampling mold levels in storm-damaged houses before, during, and after remediation to determine the effectiveness of cleanup procedures. Given the varying salinity of floodwaters that impacted homes—salt, brackish, and fresh—the team is eager to see what different types of mold appear as a result. They are also eager to discover how the long lapse in time between remediation (mostly done in the winter of 2012–2013) and reconstruction (just now beginning) has on the reappearance of mold.

**Figure:**
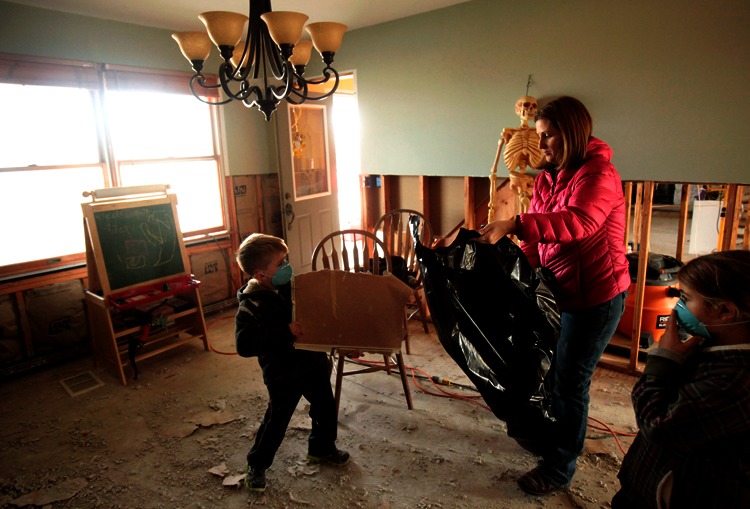
A family in Union Beach, New Jersey, cleans up a room in their home, 18 November 2012. Union Beach is a tiny borough—just 2,600 houses, 85% of which were flooded by Hurricane Sandy. The town is also coping with the loss of four firehouses, four fire trucks, an ambulance squad, and 14 police cars, plus the flooding of a K–8 school.^32^ In this and many other Jersey shore towns, where permanent populations usually number less than 10,000, locally available resources have been overwhelmed; support from county and state governments, plus volunteers, is essential to response and recovery. © Aristide Economopoulos/Star-Ledger/Corbis

“After structures undergo repairs, how will residents know if two to three months later there will be no mold regrowth or residual chemicals present?” Lioy says. “There is no program for postrepair testing. It comes down to residents making an assumption that it is safe to live in their house.”

## Resilience

On Interstate 95, just north of Newark Liberty International Airport, there is an electronic billboard that flashes one word—“RESILIENCE.” Repeated in countless conversations, newspaper, and magazine articles about the storm, the word refers to the goal to build back after the storm in a way that will enable New Yorkers and New Jerseyites to better survive future storms.

Scientists debate whether or not Sandy was caused, or at least worsened, by climate change,^19^ but there is little debate that sea levels are rising along the Atlantic seaboard. In 2012 researchers from the U.S. Geological Survey reported that sea level along the 600-mile stretch from Cape Hatteras, North Carolina, to Boston, Massachusetts, rose by 1.97–3.80 mm per year since 1990, or three to four times the global average. Extrapolating from these data, the researchers estimated that sea level along this “hot spot” could rise 20–29 cm between 1990 and 2100.^20^

In the face of this evidence, some argue that humans must retreat from the shoreline or face repeated death and destruction. New York governor Andrew Cuomo has asked the state to allocate $400 million to buy out homes wrecked by Sandy, demolish them, and restore the land as undeveloped coastline. As of this writing, that bill had not passed. New Jersey has a buyout program known as Blue Acres, targeted to floodprone properties across the state, funded at between $12 million and $50 million per year.^21^ In addition to using Blue Acres funds, Governor Christie has committed at least $250 million in federal Hazard Mitigation Grant Program^22^ dollars to buy out properties affected by Hurricane Sandy.

In January 2013 President Barack Obama signed into law the Disaster Relief Appropriations Act, which provides $16 billion in Community Development Block Grant Disaster Relief funds to repair and restore areas affected by Hurricane Sandy. New York City has submitted to the Department of Housing and Urban Development its Partial Action Plan A, which divides the city’s initial allocation of $1.77 billion among housing recovery, business recovery, infrastructure and other city services, and increased resilience against future disasters in the neighborhoods hardest hit by Hurricane Sandy.^23^ New Jersey has submitted a proposal that focuses its initial allocation of $1.83 billion on reconstruction, rehabilitation, and elevation of damaged homes, and on supporting businesses in damaged communities through grants, loans, and a tourism marketing campaign.^24^

In the meantime, communities along the Jersey shore and Long Island are building or rebuilding dune systems to lessen the impact of future storms. FEMA has drawn up new floodplain maps and established new Base Flood Elevations that call for homes in these areas to be elevated 8 feet above the floodplain.^25^ However, the cost of meeting these requirements and confusion about how local governments will interpret them is causing many homeowners to hold off on rebuilding.

“The small town organization that defines the Jersey shore is both a blessing and a curse,” says Buckley. “Each town had its own unique flooding situation—some ocean-driven, some river, some lake, and some mixed. These hazards are being addressed locally by people who understand the local geography and are eager to help each other as best they can, neighbor to neighbor. Unfortunately, they need more help. The mayor of a town of two thousand residents does not have the same resources or carry the same weight as a mayor of a city of eight million.”

For densely populated shoreline communities, many of which are dominated by apartment buildings, it may be unrealistic to think that owners will either abandon their buildings in significant numbers or raise them 8 feet in the air. Hoboken mayor Dawn Zimmer wants federal agencies to pay for “a more universal solution”—building permanent walls around those parts of the city where storm surge is likely to come from. Zimmer also wants the city to be able to disconnect from the electrical grid when power goes out and transfer to its own minigrid powered by a mix of diesel, solar, wind, and natural gas.^26^

In short, there are no easy solutions to dealing with sea level rise and storms in the New York metropolitan area. In the meantime, the focus remains on getting people back into their homes and apartments, restoring businesses and community infrastructure, and opening beaches to the summer tourists.

## Mental Health Considerations

**The Unseen Effects of Disaster**

Hurricane Sandy weakened what was already considered to be a fragile mental health care system in New York City. Before Sandy hit, metro area hospitals were already struggling to meet the demand for mental health care. After the storm, the numbers of people seeking care jumped dramatically, while the ability to treat them dropped. Storm surge knocked out several of the city’s largest psychiatric hospitals, disrupted outpatient services, and flooded scores of nursing homes (including several in the Rockaways) where many mentally ill people had found housing of last resort.^33^

According to The New York Times, Beth Israel Medical Center in Lower Manhattan saw a 69% increase in psychiatric patients in November, far more than it could handle. Maimonides Medical Center, in Brooklyn, reported a 56% increase in psychiatric emergency room visits in the month following the storm. Clergy for churches in New Brighton reported mentally ill people showing up at church rectories begging for socks and underwear.^33^

“The dominoes start falling backwards,” Yves Ades, chief operating officer of the nonprofit Services for the UnderServed, told the Times regarding the effects of the storm on the area’s mental health system. “It was always a strained system, but it was functioning. Now, it’s breaking.^33^

In response to the widespread mental anguish caused by Sandy, FEMA and other organizations have set up crisis hotlines that people can call to get counseling. FEMA also funded Project Hope, a crisis counseling program of the New York State Office of Mental Health that serves residents in New York City and four other counties. Programwide, Project Hope has hired, trained, and deployed 669 crisis counselors, 371 of whom work in the areas impacted by Sandy, to provide emotional support, education, counseling (individual, group, and family), and group public education, according to program spokeswoman Caroline Burwell. As of March 15, approximately 107,000 New Yorkers have been reached by Project Hope. Burwell adds that LIFENET is a confidential 24-hour referral hotline (1-800-LIFENET) that has been contracted to match Hurricane Sandy victims with a Project Hope provider agency in their community.
